# Reasons for Breastfeeding Cessation in the First Year after Childbirth in Lithuania: A Prospective Cohort Study

**DOI:** 10.3390/medicina56050226

**Published:** 2020-05-09

**Authors:** Viktorija Zitkute, Vilija Snieckuviene, Jolita Zakareviciene, Aurelija Pestenyte, Vaidile Jakaite, Diana Ramasauskaite

**Affiliations:** Institute of Clinical Medicine, Clinic of Obstetrics and Gynaecology, Faculty of Medicine, Vilnius University, LT-03101 Vilnius, Lithuania; snieckuviene@gmail.com (V.S.); jolita.zakareviciene@santa.lt (J.Z.); aurelija.pestenyte@gmail.com (A.P.); vaidile.jakaite@gmail.com (V.J.); diana.ramasauskaite@santa.lt (D.R.)

**Keywords:** breastfeeding cessation, breastfeeding rate, lactation, maternal leave

## Abstract

*Background and objectives:* to identify the main reasons of breastfeeding cessation in Lithuania and if there is a link between the length of maternity leave and breastfeeding cessation. *Materials and methods:* a prospective questionnaire study was conducted in a tertiary hospital from 2016 to 2017. The sample size included 449 women. *Results:* a total of 41% (*n* = 123) of respondents weaned off by 6 months after birth, and 57.8% (*n* = 173) between 6 months and 1 year. During the first few days after delivery, mothers did not breastfeed their infants mainly due to shortage of milk (*n* = 10; 40%) or separation from their baby due to infant health problems (*n* = 12; 48%) (*p* < 0.0001). Mothers who did not breastfeed during the first days after birth more often did not start breastfeeding later at home (*p* = 0.001). Going back to work was not a significant factor in weaning off. *Conclusions*: breastfeeding initiation and practice during the first few days after birth has a significant impact on the further commitment for full breastfeeding. Additionally, a perceived lack of support and help from both doctors and midwives influences a woman’s decision to choose not to breastfeed.

## 1. Introduction

Breastfeeding is undeniably beneficial for the infant and is very important for the mother’s health. Although most mothers can breastfeed, according to research by The World Health Organization (WHO), 1%–2% cannot produce enough milk due to a specific pathology of the body [1]. This can be caused by both the physical and emotional state of the mother. The most critical period for cessation of breastfeeding is 1–4 months post-birth, where about 10%–20% of babies are weaned. After the fourth month, about 3%–6% of mothers stop breastfeeding per month [1,2].

Despite the positive attributes and an increasing body of evidence supporting the benefits of breastfeeding, only 38% of infants around the world are exclusively breastfed for the recommended 6 months after delivery [3]. Although breastfeeding has increased in all regions of the world, global progress has halted. For this reason, in 2012, The World Health Organization’s Assembly set a common goal to increase the number of breastfeeding infants up to the age of 6 months by at least 50% by the year 2025 [4].

According to studies in the United Kingdom, the prevalence of lactation in many European countries is low, but rising slowly. The countries with the lowest breastfeeding practice for infants up to the age of 6 months are the UK (34%), France (23%), Germany (40%), Spain (24.7%), Switzerland (14%), and the Netherlands (20%) [5,6,7,8]. High lactation prevalence is most noticeable in the Scandinavian countries (80% in Norway and 65% in Sweden) [9,10].

Breastfeeding initiation has great influence for productive breastfeeding practice. The WHO recommends breastfeeding to start within the first hour after birth to ensure that the baby receives colostrum, which is rich in protective factors, nutrients, etc. Recent evidence suggests that skin-to-skin contact between mother and baby shortly after birth helps to initiate breastfeeding and increases the likelihood of exclusive breastfeeding at 1–4 months postpartum, as well as the overall duration of breastfeeding.

The discussion of the length of maternity leave influencing the duration of breastfeeding was raised several decades ago [11]. Women living in countries with short maternity leave have a tendency to stop breastfeeding before or shortly after they return to work.

Of 193 countries in the United Nations, the United States is one of three, along with Oman and Papua New Guinea, that do not offer paid maternity leave [12]. Although the United States does not guarantee paid maternity leave, employers may provide paid leave if they choose. There are three states in the United States that do provide paid maternity leave: California, New Jersey, and Rhode Island [13]. Countries that offer paid maternity leave include Mexico (12 weeks), the United Kingdom (40 weeks), India (26 weeks), Chile (6 weeks before birth, 12 weeks after), Canada (1 year), and China (14 weeks) [14].

In several countries, mothers are able to share infant care with their husbands because paternity leave is available. For example, in Slovenia, fathers have 12 weeks of 100% paid paternity leave; in Sweden, 480 days of 80% paid paternity leave; and in Norway, 49 weeks of 100% or 59 weeks of 80% paid paternity leave [15].

Nordic countries are recognized for their generous paid leave policies, but the Baltic States, with Lithuania in the lead, offer the most generous leave of all countries. Due to a low birth rate and decades of negative population growth, Lithuania offers one of the longest paid leaves for both mothers and fathers in the world. Women who have been employed or who have paid social security insurance can benefit from a pregnancy and childbirth leave at a 100% salary-equivalent allowance paid for 18 weeks, starting at the 30th week of gestation until 2 months after birth [16]. A father has the right to take 100% paid paternal leave for the first month after birth. After an infant turns 2 months old, either new mothers or fathers, including caregivers and parents adopting their child, might choose to have 100% salary-equivalent leave until the child reaches the age of 1 year. Instead, if the insured chooses to have maternal/parental leave payments until the child reaches the age of two, the amount of this allowance from childbirth until the child reaches 1 year is 70% salary equivalent, and 40% until the child reaches 2 years of age.

Keeping in mind that Lithuanian mothers have the possibility to have one of the longest paid maternity leaves in the world and, therefore, hypothetically breastfeed infants longer, our study aim was to identify the main reasons of breastfeeding cessation in Lithuania and to investigate the influence of parental and infant characteristics on breastfeeding from birth to 12 months of age, as well as to explore if there is a link between the length of maternity leave and breastfeeding cessation.

## 2. Materials and Methods

A prospective observational cohort study was conducted at the tertiary referral Obstetrics and Gynecology Centre of Vilnius University Hospital Santaros Clinics from 2016 to 2017. This maternity hospital did not have a Baby Friendly Hospital Initiative Certificate. The study was conducted with the permission of the Vilnius Regional Biomedical Research Ethics Committee (Protocol No. 158200-16-827-342). All women who agreed to participate in the study signed informed consent.

Interviews, questionnaires, and medical records from maternal and neonatal health histories were used to collect data on maternal antenatal and intrapartum, family, neonatal, and medical factors. Original questionnaires were prepared for the study. The content of the questionnaires was validated by experts in breastfeeding, maternal, and child health. A pilot study was undertaken with 40 participants.

We calculated the research sample size with a significance level of 95% and a power of 80%. According to 2014 statistics, a total of 12,150 women gave birth in the region of Vilnius. Using the sample calculation formula, we needed at least 378 women.

**Figure 1:**
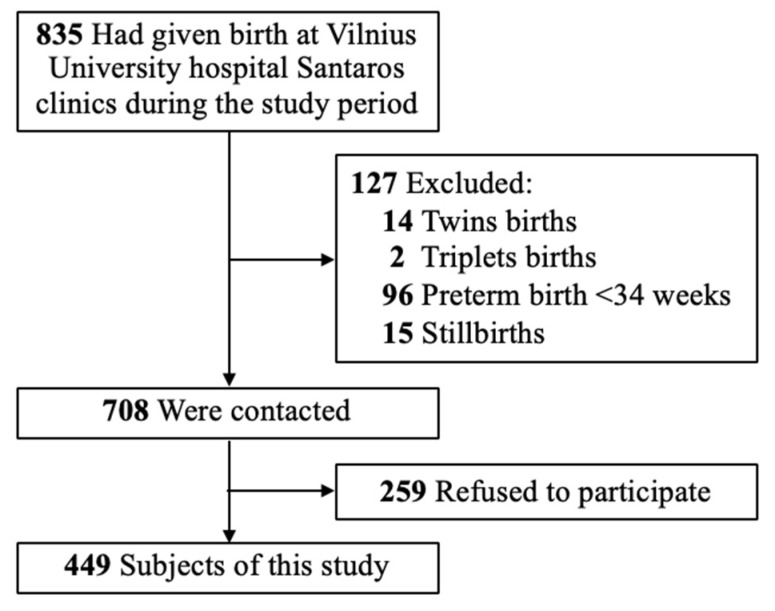
Subject selection.

Our criteria for inclusion in the study is as follows: maternal age of 18–45 years and birth after a single pregnancy ≥ 34 weeks of gestation, regardless of their parity status or delivery mode (including vaginal, instrumental, and cesarean section deliveries). Criteria for exclusion: refusal of the mother to continue to participate in the study or being unreachable by telephone; multiple pregnancies; preterm births < 34 weeks; women who delivered a stillborn baby, or women who experienced a newborn/infant death. The subject selection is described in Figure 1.

The study was divided into two stages: at the first stage, the researchers collected data on the 2nd–4th day after birth in the hospital based on the inclusion criteria (the researchers visited postpartum wards and asked if woman would agree to participate in the study); at the second stage, data were collected by the same researchers through telephone interviews at 6 weeks and 3, 6, and 12 months postpartum.

The statistical analysis of data was performed using IBM SPSS Statistics 23.0 and Microsoft Office Excel 2016. The Shapiro–Wilk test was performed on quantitative data to determine the normality distribution of the data. If the distribution of normality was met, the data were expressed as mean and standard deviation (SD) and were analyzed by the independent-samples *t*-test. If not, the data were expressed as median (interquartile range), and the Mann–Whitney *U* test was used for the comparisons. Qualitative data are expressed as the number of respondents and percentages (%), analyzed by the Pearson Chi-squared test. Differences are considered statistically significant at *p* ≤ 0.05.

## 3. Results

**Figure 2:**
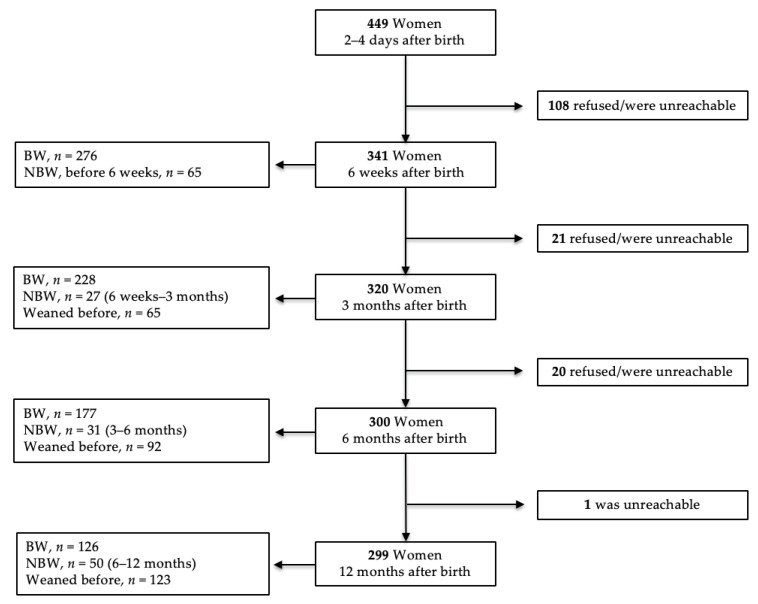
Study population. BW, breastfeeding women; NBW, non-breastfeeding women.

Data analysis was performed by dividing women in two groups: breastfeeding women (BW) were defined as any who were breastfeeding at birth (2–4 days after birth), and at 6 weeks and 3, 6, and 12 months; the non-breastfeeding women (NBW) group consisted of those who weaned off before 6 weeks, between 6 weeks and 3 months, between 3 and 6 months, and between 6 and 12 months. The number of women interviewed in the study for each period is shown in the Figure 2.

### 3.1. Study Population Basic Information

At the first stage of the study, 2–4 days after childbirth, 66.8% of subjects were primiparous women and 33.2% multiparous. There were no significant differences in age, marital status, or social factors such as residential area or educational level between women who initiated breastfeeding and those who had not started it yet.

The majority of mothers (*n* = 236; 52.7%) planned to breastfeed a baby for 1 year, and 107 (23.9%) for half of a year, while 35.9% (*n* = 161) of the subjects did not report any specific planned breastfeeding period. Almost one third of women (*n* = 139; 30.9%) answered that they had substituted breastfeeding with infant formula and 33.4% (*n* = 150) of mothers gave a pacifier to newborns.

From women who had undergone cesarean section, there were 45.8% who had not initiated breastfeeding by 2–4 days after birth, while in natural birth group, 20.8% (*p* = 0.016). During the first few days after delivery, mothers did not breastfeed their infants mainly due to shortage of milk (*n* = 10; 40%) or separation from their baby due to infant health problems (*n* = 12; 48%) (*p* < 0.0001). Notably, mothers who did not breastfeed during the first days after childbirth more often did not start breastfeeding later at home (*p* = 0.001).

### 3.2. Breastfeeding at Home

#### 3.2.1. Six Weeks after Giving Birth

A total of 341 women participated in the follow-up telephone interview; 276 (81%) of them continued with at least some breastfeeding, 188 (55.1%) were breastfeeding exclusively, and 65 (19%) had weaned off by the 6-week mark.

The NBW group before 6 weeks was composed of women who had a significantly lower education level, were more frequently unmarried, lived in the countryside, and smoked during pregnancy or were currently smoking (Table 1). NBW stated that they weaned off due to a shortage of milk (*n* = 41; 63%); problems with infant (7; 10.9%) or mother health issues (10; 15.2%); and from the decision of the mother to not breastfeed (6; 8.7%). The most common reason for mothers who had breastfeeding problems was that they experienced nipple problems (pain, tearing, cracking, bleeding)—102 women (36.8%) from the BW group vs. 15 women (26.3%) from the NBW group (*p* = 0.085) (Table 2).

Although the majority of the women in the NBW group (75.5%) claimed that they had been breastfeeding infants on demand before they weaned off, the majority of subjects (83.9%) gave a pacifier or other liquids (water, tea) when the baby got fussy instead of offering a breast. In addition, they had lower confidence about their breastfeeding knowledge and complained about lack of support from family members and medical staff. An analysis showed that women who weaned off earlier (34; 53.2%) showed a statistically significant likelihood to feel confusion, sadness, and guilt for not breastfeeding (*p* = 0.029).

Three women (4.6%) from the NBW group at 6 weeks mentioned that they had returned to work or had resumed their studies vs. 28 women (10.1%) in the BW group (*p* = 0.044) (Table 2).

#### 3.2.2. Three Months after Giving Birth

During this period, 228 women were still breastfeeding (*n* = 191; (83.7%) exclusively, and 37 (16.3%) were partially breastfeeding, while 27 (8.4%) had stopped breastfeeding between 6 weeks and 3 months). The NBW group between 6 weeks and 3 months was composed of subjects who had significantly higher body mass index (BMI), nipple problems, and less support from family and medical staff. The NBW group had shorter skin-to-skin length compared to BW group (*p* = 0.021). Women from cities weaned off earlier (*p* = 0.011) (Table 1 and Table 2).

The NBW group between 6 weeks and 3 months after delivery stated that they weaned off due to a shortage of milk (20; 74.1%), the infant’s refusal of the breast (3; 11.1%), or the mother’s health issues (6; 22.2%) (*p* < 0.0001). A little more than half of NBW (*n* = 14; 51.9%) felt sadness, guilt, and disappointment.

Additionally, at this period, four women from the NBW group started complementary feeding (at an average of 11.5 weeks), while none from the BW group had done so. The NBW group was slightly more likely to give their infants additional fluids and a pacifier (63% and 92.6%, respectively). Three women from the NBW group (11.1%) mentioned that they had returned to work or had continued their studies vs. three women (1.3%) in the BW group (*p* < 0.0001) (Table 2).

#### 3.2.3. Six Months after Giving Birth

According to the data, 177 women were still breastfeeding (*n* = 117, (39%) exclusively and 60 (20%) partially). We also found that 31 (10.3%) women weaned off their babies between 3 and 6 months.

Older maternal age and lower BMI were the variables found to be significantly associated with continued breastfeeding (Table 1). The NBW group continued to show higher intake of additional fluids and greater pacifier use (93.5% and 90.3%, respectively). The NBW group started complementary feeding at median of 20 (8) weeks and the BW group started solids at median of 22 (4) weeks (*p* = 0.321) (Table 2). Between 3 and 6 months after delivery, women noted that they weaned off due to a shortage of milk (*n* = 22; 71%), the baby refusing the breast (*n* = 5; 16.1%), and the mother feeling breastfeeding to be inconvenient or getting bored with breastfeeding (*n* = 8; 25.8%). Women from the countryside breastfeed longer (*p* = 0.045). It was found to be of statistical significance that the NBW group at this period more often felt psychologically good, and claimed that they do not mind stopping breastfeeding (*n* = 24; 77.4%) (*p* < 0.0001).

Three women (9.7%) of the NBW group mentioned that they had returned to work, while five women (2.8%) reported the same in the BW group (*p* = 0.028).

#### 3.2.4. One Year after Childbirth

The last telephone survey revealed that 50 women (16.7%) weaned off. In total, 173 (57.9%) women were not breastfeeding after 1 year. At this time, a pacifier had been given by 35 women (70%) (*p* = 0.016).

The most common reason to wean off between 6 and 12 months was the decision by the mother to do so (*n* = 18, 36%). Six women weaned off due to their health disorders (12%) or their infant’s refusal of the breast (*n* = 7; 14%). During this period, NBW felt good about their decision and claimed that it was time to end breastfeeding (*n* = 41; 82%). Comparing between the groups, 11 (22%) of NBW group had already returned to work, while 32 (25.4%) women had returned to work from the BW group (*p* = 0.002) (Table 2).

### 3.3. Discussion

According to our study, women who had undergone cesarean section or had infants with health problems were significantly more likely to have never initiated breastfeeding during their stay in hospital, and even more often did not begin breastfeeding at home. These women were more vulnerable due to physical and emotional responses to surgery, as well as infant health, behavior, or separation. In 2016, Hobbs et al. did a prospective cohort study, which showed that women who had experienced a planned or emergency cesarean section were more likely to have had an unsuccessful first breastfeeding attempt, were unable to breastfeed their baby within the first 24 h, or did not breastfeed upon leaving the hospital [17]. Based on the literature review, infrequent breastfeeding, limited mobility of the mother in the early days after surgery, and postoperative pain are the main factors that have a negative impact on the breastfeeding experience [18].

In a 2008 study conducted in Japan, it was found that if the first breastfeeding occurred within 120 min postpartum, the event significantly influenced the duration of breastfeeding in the hospital and at home [19]. A Swedish study showed that women’s early contact with their newborn for at least 20 min after birth decreases breastfeeding problems and increases breastfeeding duration [9]. All of these data concur with our study, showing that if initiation of breastfeeding was delayed or the contact was shorter, the mother had more breastfeeding problems and weaned off earlier.

The relatively high number of non-breastfeeding mothers is largely determined by psychosocial reasons and inadequate or unqualified care. In a systematic review by Beake et al., they identified a few interventions that are specifically targeted to increase breastfeeding after cesarean section: immediate or early skin-to-skin contact, and education and support of breastfeeding [20]. Guidelines by the WHO state that keeping the mother and infant together for at least the first hour after birth leads to improved initiation and duration of breastfeeding [21].

All women, and especially those from vulnerable groups, must have access to a lactation consultant and a psychologist during their hospital stay. More importantly, mothers should have access to proper postpartum home visits and primary healthcare facilities, which present ideal opportunities to provide additional breastfeeding assessments and support to overcome any difficulties these women may experience [22,23]. Teaching and promoting natural feeding boosts the initiative to breastfeed a baby for up to 6 months [24,25,26]. By improving these very important care steps mentioned above, we could eliminate the main obstacles faced by mothers who do not breastfeed in this early time after childbirth.

Our research highlighted that the most sensitive periods of weaning off are after the first 6 weeks after childbirth. The main reason to stop breastfeeding after 6 weeks was due to a shortage of milk [27,28]. The problem of breast milk shortage, which we could call physiological, is largely determined by the time the infant first suckles, and how often he/she breastfeeds. According to a study conducted in Lithuania in 2009, the following medical factors have implications for lactation: late breastfeeding initiation, infant separation from mother, short initial lactation stimulation, absence of skin-to-skin contact, pacifier use, and early introduction of additional food [29]. There is also a significant relationship between weaning off and swollen breasts, wounded/painful nipples, and anxiety due to the appearance of breasts. Another important factor is that it is better to breastfeed according to the infant’s needs throughout the entire day [30], which was also proved by our study (*p* < 0.0001).

Some of the mothers from our study mentioned that they had lower confidence about their breastfeeding knowledge, and complained about lack of support from family members and medical staff during the first weeks after childbirth. In an Iranian study, the main causes of weaning off that women presented were the physician’s recommendation, insufficient breast milk, and family recommendation [27]. Therefore, social support for breastfeeding from a woman’s family and medical staff has been implicated as an important factor in influencing the choice and duration of breastfeeding [31] Moreover, research in Western societies indicates that the support of fathers is critical to breastfeeding success and is identified as one of the strongest factors associated with women’s willingness to breastfeed [2]. Based on research by Stuebe et al., physicians seeing women for either mood disorders or lactation difficulties should be aware of this and should assess women with breastfeeding problems for depression, as well as should refer women with depressive symptoms for breastfeeding support from an experienced provider [32].

Another sensitive period of weaning off is from 6 months to 1 year. Based on our study, this was the time when mothers stopped breastfeeding mainly due to their own decision, because it had become difficult due to their daily lives or they were bored. Support during this period is important, but we can also see that a mother’s education and intention to breastfeed plays very big role too. Smith et al. noticed that a mother’s education is a characteristic that positively influences breastfeeding rates at 6 and 12 months [30]. In our research, they clearly stated that by that time, they had become confident and proud of their decision, which means that either mothers do not care about the benefits of breast milk or they lack knowledge about the benefits.

It is believed that returning to work is one of the most common causes of breastfeeding cessation. However, before 6 weeks, less than 10% of BW and less than 5% of NBW returned to work. Even though the numbers of women of returning to work are low at this time period, this might be crucial to establish adequate lactogenesis [33]. Women did not mention that their intention to wean off was due to returning to work. A survey conducted in the United States revealed that each week of maternity leave taken increased the breastfeeding duration by about half a week [34].

The first limitation in this study was the recall bias. Postpartum mothers were interviewed over the phone at the end of 6 weeks and 3, 6, and 12 months after birth, so usually, if it was hard to reach them, they refused to speak or participate in the study. Due to the decline in the sample size of patients in 6–12 months, we may consider the results of this period to be not strongly statistically significant, but still emphasizing/highlighting certain aspects. The second limitation was the sample selected from the one Vilnius hospital, which can affect the generalization of the results to all Lithuanian postpartum mothers. The third limitation was the fact that each postpartum mother was asked closed-ended questions regarding the difficulties of her practicing breastfeeding from a pre-prepared list, which might not have captured other difficulties not present in the list.

Finally, we believe that the size of the studied sample gives significance to these results, because the whole target population was covered. Establishing which groups are at high risk, such as being a single mother or having a low educational level, should lead to the implementation of educational strategies early during pregnancy. In the same way, non-healthy habits such as alcohol and cigarette smoking during pregnancy are subjects that must be discussed. Furthermore, studies to define the role of education programs at the initiation and duration of breastfeeding, as well as to analyze the effect of family and medical stuff support, must be carried out.

## 4. Conclusions

Our study indicates that going back to work was not a significant factor in weaning off from breastfeeding. At present, Lithuanian working mothers can breastfeed their babies longer due to the high support of their family members, the fathers’ opportunity to have paternity leave, and feeding babies with the mothers’ milk.

Midwives’ and doctors’ help during the first days at hospital after birth is very relevant and provides a greater opportunity for future breastfeeding at home. Our research revealed that the most sensitive periods of weaning off are the first 6 weeks after childbirth and from 6 months to 1 year. The main differences between the two time frames are the mother’s opinion and her attitude toward weaning off. Mothers have a long-standing view that breastfeeding after 6 months is not very important. That is why we believe that during these periods, mothers should contact their consultant for lactation.

## Figures and Tables

**Table 1:** Participants’ characteristics.

	BW until 6 Weeks (*n* = 276)	NBW until 6 Weeks (*n* = 65)	*p*	BW between 6 Weeks and 3 Months (*n* = 228)	NBW between 6 Weeks and 3 Months (*n* = 27)	*p*	BW between 3 and 6 Months (*n* = 177)	NBW between 3 and 6 Months (*n* = 31)	*p*	BW between 6 and 12 Months (*n* = 126)	NBW between 6 and 12 Months (*n* = 50)	*p*
**Age, mean (SD), years**	30.8 (4.6)	30.2 (6.3)	0.474	31 (4.5)	30.3 (5.1)	0.362	31.4 (4.5)	28.5 (4.4)	**0.001**	31.5 (4.4)	30.4 (3.5)	0.092
**Education level, *n* (%)**												
secondary	24 (8.7)	24 (36.4)	**<0.0001**	10 (6.1)	2 (7.7)	0.506	24 (13.5)	5 (16.2)	0.072	11 (8.7)	6 (10.2)	0.331
>secondary	253 (91.3)	42 (63.6)		218 (93.9)	25 (92.3)		153 (86.5)	26 (83.8)		115 (92.2)	44 (89.8)	
**Marital status, *n* (%)**												
Married	249 (89.9)	44 (66.7)	**<0.0001**	145 (66.5)	24 (88.9)	0.559	143 (80.8)	29 (94.3)	0.211	106 (84.1)	46 (92.0)	0.262
Single	28 (10.1)	22 (33.3)		73 (33.4)	3 (11.1)		34 (19.2)	2 (5.7)		20 (15.8)	4 (8.0)	
**Residential area, *n* (%)**												
city/town	222 (82.3)	44 (67.7)	**0.011**	134 (61.4)	18 (66.6)	**0.011**	130 (73.4)	25 (80.6)	**0.045**	117 (92.9)	40 (80.0)	0.466
countryside	55 (17.7)	22 (32.3)		84 (38.5)	9 (33.3)		47 (26.6)	6 (19.4)		9 (7.1)	10 (20.0)	
**Weight gain during pregnancy, mean (SD), kg**	14.9 (4.6)	15.2 (5.9)	0.371	14.8 (4.5)	14.9 (5.7)	0.910	14.8 (4.5)	16.3 (4.7)	0.105	14.7 (4.7)	13.8 (4.1)	0.217
**BMI, median (IQR)**	21.8 (4.3)	22.8 (5.3)	0.053	21.8 (4.8)	23.8 (6.7)	**0.013**	22.1 (4.6)	21.6 (5.8)	**0.003**	22.1 (5.0)	21.4 (4.1)	0.626
**Smoking during pregnancy, *n* (%)**	3 (1.1)	7 (10.6)	**0.001**	1 (0.4)	1 (3.7)	0.261	1 (0.5)	0 (0)	0.823	1 (0.8)	0 (0)	0.766

BW, breastfeeding women; NBW, non-breastfeeding women; BMI, body mass index; IQR, interquartile range. Data expressed with mean and standard deviation (SD) were analyzed by the independent-samples *t*-test. For the data expressed as median (interquartile range), the Mann–Whitney *U* test was used for the comparisons. Qualitative data were analyzed by the Pearson Chi-squared test.

**Table 2:** Factors influencing the cessation of breastfeeding during the first year after delivery.

	BW until 6 Weeks (*n* = 276)	NBW until 6 Weeks (*n* = 65)	*p*	BW between 6 Weeks and 3 Months (*n* = 228)	NBW between 6 Weeks and 3 Months (*n* = 27)	*p*	BW between 3 and 6 Months (*n* = 177)	NBW between 3 and 6 Months (*n* = 31)	*p*	BW between 6 and 12 Months (*n* = 126)	NBW between 6 and 12 Months (*n* = 50)	*p*
**Caesarian section, *n* (%)**	58 (21)	18 (27.3)	0.174	30 (13.1)	6 (22.2)	0.393	33 (18.6)	13 (41.9)	**0.032**	33 (26.2)	5 (10.0)	0.054
**Plans unlimited breastfeeding length, *n* (%)**	84 (31.1)	31 (49.2)	**0.006**	112 (49.1)	20 (74.1)	0.411	49 (27.6)	10 (32.2)	0.515	46 (36.5)	14 (28.0)	0.465
**Family support, *n* (%)**	241 (87.3)	32 (62.7)	**<0.0001**	150 (65.7)	14 (51.8)	**0.014**	149 (84.1)	10 (32.2)	0.562	115 (91.2)	23 (46.0)	0.194
**Medical staff support, *n* (%)**	208 (76.5)	26 (49.1)	**<0.0001**	133 (58.3)	12 (44.4)	**0.039**	134 (75.7)	10 (32.2)	0.456	102 (80.9)	22 (44.0)	0.128
**Healthy newborn, *n* (%)**	205 (79.8)	44 (72.1)	0.131	124 (54.3)	19 (79.2)	0.507	125 (70.6)	25 (80.6)	0.373	120 (95.2)	39(78.0)	0.473
**Any skin-to-skin after birth, *n* (%)**	179 (65.8)	37 (57.8)	0.146	110 (48.2)	15 (57.7)	0.223	105 (59.3)	21 (67.7)	0.328	104 (82.5)	36 (73.5)	0.275
**Uninterrupted skin-to-skin before first breastfeeding, *n* (%)**	51 (18.9)	7 (11.3)	0.105	36 (15.7)	5 (19.2)	0.480	35 (19.7)	4 (12.9)	0.116	35 (28.0)	9 (18.4)	0.319
**Skin-to-skin length, median (IQR), min**	7 (13)	5 (9.5)	**<0.0001**	5 (13)	4 (10.5)	**0.021**	5 (13)	5 (10.3)	0.328	5 (14)	10 (15)	0.516
**What minute after birth started to breastfeed, median (IQR), min**	60 (91)	60 (330)	0.056	60 (90)	60 (90)	0.506	60 (90)	60 (60)	0.439	60 (90)	60 (23)	0.423
**Get extra fluid, *n* (%)**	34 (12.3)	30 (47.6)	**<0.0001**	42 (18.4)	17 (63.0)	**<0.0001**	89 (50.2)	29 (93.5)	**0.001**	115 (91.2)	46 (93.9)	0.555
**Pacifier use, *n* (%)**	182 (65.9)	52 (83.9)	**0.003**	87 (38.1)	25 (92.6)	**<0.0001**	89 (50.2)	28 (90.3)	**<0.0001**	82 (65.0)	35 (70.0)	**0.016**
**Started complementary food, median (IQR), weeks**	-	-	-	-	-	-	22 (4)	20 (8)	0.321	22 (4)	22 (4)	0.189
**Sore nipples, *n* (%)**	102 (36.8)	15 (26.3)	0.085	11 (4.8)	5 (21.7)	**0.032**	15 (4.5)	4 (3.2)	0.726	6 (4.7)	1 (2.0)	0.609
**Lactostasis, *n* (%)**	45 (16.2)	6 (10.5)	0.188	15 (6.5)	2 (7.4)	0.648	15 (8.4)	2 (6.4)	0.154	5 (3.9)	1 (2.0)	0.685
**Thinks that does not have enough knowledge about breastfeeding, *n* (%)**	24 (9.0)	20 (38.5)	**<0.0001**	8 (3.5)	2 (7.4)	0.329	4 (2.2)	3 (9.7)	**0.048**	5 (3.9)	5 (10.0)	0.115
**Actual smoking, *n* (%)**	2 (0.7)	8 (12.9)	**<0.0001**	0 (0)	1 (3.7)	0.141	1 (0.5)	0 (0)	0.827	3 (2.3)	2 (4.0)	0.827
**Going to work, *n* (%)**	28 (10.1)	3 (4.6)	**0.044**	3 (1.3)	3 (11.1)	**<0.0001**	5 (2.8)	3 (9.7)	**0.028**	32 (25.4)	11 (22)	**0.002**

BW, breastfeeding women; NBW, non-breastfeeding women; BMI, body mass index; IQR, interquartile range. Data expressed with mean and standard deviation (SD) were analyzed by the independent-samples *t*-test. For the data expressed as median (interquartile range), the Mann–Whitney *U* test was used for the comparisons. Qualitative data were analyzed by the Pearson Chi-squared test.
